# Enabling and Predisposing Factors for the Utilization of Preventive Dental Health Care in Migrants and Non-Migrants in Germany

**DOI:** 10.3389/fpubh.2017.00201

**Published:** 2017-08-14

**Authors:** Patrick Brzoska, Fabian Erdsiek, Dorothee Waury

**Affiliations:** ^1^Chemnitz University of Technology, Faculty of Behavioral and Social Sciences, Institute of Sociology, Epidemiology Unit, Chemnitz, Germany

**Keywords:** migrants, oral health, disparities, utilization, Germany

## Abstract

**Background:**

In many European countries including Germany, migrants utilize preventive services less frequently than the majority population. This is also true for the utilization of dental checkups. Little is known about which demographic, social, behavioral, and health-related factors influence the decision of migrants to seek preventive dental health care and how these factors differ from those in non-migrants. The aim of the present study was to examine the role of these factors among migrants and non-migrants residing in Germany.

**Methods:**

Data from cross-sectional national health surveys are used, providing information on preventive dental health behavior from *n* = 41,220 individuals, of which 15.0% are migrants. Andersen’s Behavioral Model of Health Services Use is the conceptual framework of the investigation. Multiple logistic regression models were applied to examine the role of different predisposing and enabling factors. Interaction terms were included in order to examine whether determinants differ between migrants and non-migrants. Average marginal effects (AMEs) are reported in addition to odds ratios (ORs) as measures of effect size which are robust against bias arising from unobserved heterogeneity.

**Results:**

Migrants are at an about 36% lower chance of utilizing regular dental checkups than non-migrants [OR = 0.64 (95% confidence interval, 95% CI: 0.61, 0.68); AME = −0.081 (95% CI = −0.093, −0.069)]. Differences are partly explained by the influence of demographic, social, behavioral, and health-related factors [adjusted OR = 0.69 (95% CI: 0.64, 0.73); AME = −0.065 (95% CI = −0.076, −0.053)]. Younger age, being male, lower socioeconomic status, a non-statutory health insurance, not living in a relationship, living in the Western part of Germany and in an urban setting, and poor limited social support were associated with a lower chance of utilizing regular dental checkups. Interaction effects could be observed for age and for the type of health insurance.

**Discussion:**

The study identifies different enabling and predisposing factors that are relevant for the utilization of dental checkups among the population in Germany, some of which differ between migrants and non-migrants. Differences are particularly pronounced for younger ages. This differs from findings on other preventive services where older migrants tend to be more disadvantaged. Additional explanatory factors such as barriers that migrants experience in the dental health care system need to be considered in order to implement patient-oriented services and to reduce disparities in access to dental prevention.

## Introduction

In many European countries including Germany, large proportions of the respective populations are migrants (1). This comprises both foreign nationals and nationals of the respective countries who have an immigrant background because they or their parents immigrated from another country. In Germany, around one-fifth of the total population of 81.4 million people are migrants, totaling about 17.0 million individuals (2).

Migrants utilize preventive measures, such as screening, less frequently than the majority population of the respective host countries (3, 4). This is also true for the utilization of regular dental checkups (5–7), which can be considered an important aspect of maintaining and promoting oral health (8–12).

Studies addressing differences in health care utilization between population groups have increasingly used Andersen’s Model of Health Services Use or variations of it to identify determinant factors (13). Concerning dental services and especially dental prevention, a few studies are available that used this model to identify determinants of service use (12, 14–16). The model distinguishes between three types of individual factors that facilitate or impede access to and utilization of health care services: predisposing, enabling, and need factors (17, 18). Predisposing factors identified in the dental care setting include sociodemographic determinants such as age, sex, socioeconomic status (SES), family status, immigration status, and aspects such as health literacy and health beliefs (11, 19, 20). Enabling factors refer to individual or structural resources enabling or increasing the likelihood of service use. In dental care, this includes aspects such as income, health insurance coverage, availability of health services or regular sources of care, and means of transportation (16, 21–23). Need factors in dental care encompass indicators of objective need of health care, such as toothache, denture wearing, carious and decayed surfaces, or other indicators of oral disease, as well as perceived (subjective) need (19, 20, 22, 23). In terms of migration, previous studies—most of which, however, did not use Andersen’s model as their theoretical framework—focused on how the proportion of those not utilizing dental prevention differs between migrants and non-migrants of different age groups (6, 7, 9–12, 24–26). Little is known about which demographic, social, behavioral, and health-related factors influence the decision of migrants to seek preventive dental health care and how these factors differ from those in non-migrants. The aim of the present study was to examine the role that these factors have for the use of dental checkups in migrants and non-migrants residing in Germany. Insights can help to inform the implementation of patient-oriented services and to reduce disparities in access to dental care.

## Materials and Methods

### Data

The analysis is based on secondary data from two cross-sectional telephone surveys (“German Health Update 2009” and “German Health Update 2010”), carried out between July 2008 and July 2010 by the Robert Koch Institute, a scientific institution of the German Federal Ministry of Health (27). Data were collected by means of a random digits approach. The aim of the surveys was to inform about the health status and the health behavior of the population in Germany aged 18 years or older who lived in a private household with a landline telephone. Both surveys used a similar core set of questions which also covered the outcome of utilization of dental checkups in the 12 months prior to the interview. As the survey has only been conducted in German language, it is only representative for migrants with good German language proficiency. Data from both surveys have been pooled for the present study. The survey data collected by the Robert Koch Institute fulfils all necessary requirements and guidelines of the Federal data protection act. The telephone survey was voluntary and anonymous. Participants provided their oral informed consent before participating in the survey (27). As the study was observational (so no experiments were conducted), no further ethical approval was necessary (28). Given that patients were sampled by means of random digits dialing and that the questionnaire was administered *via* telephone obtaining a written informed consent was not feasible.

### Variables

In the analysis, we compare migrants and non-migrants. In line with the procedure in other studies (29), migrants were defined as individuals who had migrated to Germany themselves or of whom at least one parent had migrated to Germany. Since only German-speaking adults were included, the sample is not representative for migrants with low or no proficiency of the German language.

As predisposing factors according to the Andersen model, sex, age (5-year age groups treated as a continuous measure), SES [low, middle, and high; based on a measure summarizing vocational educational, occupational status, and net equivalent income (30)], and marital status (living with a partner vs. not living with a partner) were taken into account. As enabling factors according to the Andersen model, the type of health insurance (statutory, private/other), social support [poor, moderate, and strong; based on the Oslo-3 Social Support Scale (31)], the place of residence (West Germany, East Germany), and the type of residence (urban, rural) were considered. The place and type of residence were included to take into account regional differences in the availability of dental services (see Table 1 for an overview of determinants of service use included in the analyses and for a description of their measurement). The outcome of our study was utilization (yes/no) of dental checkups in the last 12 months prior to the survey based on self-reports by respondents.

**Table 1:** Potential determinants of service use included in the analyses and description of measurement.

Factors included		Measurement: questions/scales/psychometric instruments	Categories of the variables/response options
Predisposing	Age	Calculated from year and month of birth	5-year age groups (treated as a continuous measure)
	Sex	Self-reported	Male, female
	Living with a partner	Summarized indicator based on three questions: (1) Are you married? (2) If not: do you have a stable non-marital partner? (3) Do you live together with your partner/spouse?	Yes, no
	Migration status	Summarized indicator based on two questions: (1) Were you born in the area of the current Federal Republic of Germany? (2) Were both your parents born in the area of the current Federal Republic of Germany?	Migrant, non-migrant
	Socioeconomic status	Metric index measure including information on vocational training, level of education, occupational status and net equivalent income (3–21 points) (30)	Low (first quintile), middle (second to fourth quintile), high (fifth quintile)
Enabling	Social support	Oslo-3 Social Support Scale (31)	Weak, moderate, and strong
	Health insurance	Self-reported health insurance status	Statutory, private, or others
	Place of residence	Based on self-reported district and state of residence	West Germany, East Germany (including Berlin)
	Urbanity	Based on self-reported size of the city/town of residence	Urban, rural

### Analysis

Aside from a sample description stratified by migration status using chi-square tests and Wilcoxon–Mann–Whitney tests where appropriate, we used a multivariable logistic regression model to examine predisposing and enabling factors associated with the use of dental services (32). All variables were entered at once, i.e., no backward/forward selection has been performed. In order to examine whether predisposing and enabling factors differ between migrants and non-migrants, we included interaction effects between all predisposing/enabling factors and migration status one by one into the model and tested for significance. Considering that the evaluation of interaction effects based on odds ratios (ORs) may be biased because of unobserved heterogeneity (33), we calculated average marginal effects (AMEs) with their respective 95% confidence interval (95% CI) along ORs. AMEs represent differences in the probability for the occurrence of the outcome. Analyses were conducted using Stata 13 (34). As models with interaction terms are difficult to interpret, we only present AMEs of significant interactions.

## Results

Information on *n* = 41,220 subjects was available, of which 15.0% were migrants. In terms of the distribution of predisposing and enabling factors, some differences between both populations could be identified. Migrants were on average younger than non-migrants, had a lower SES, were more often insured by means of statutory health insurance instead of private health insurance, and lived more often in urban settings as well as in the Western part of Germany. The percentage of individuals reporting a lower social support was also higher among migrants. Only small differences could be observed in terms of sex ratio and the proportion of individuals who lived together with a partner (Table 2).

**Table 2:** Sample description by migration status (German Health Update 2009/2010 survey, *n* = 41,220).

Factor	Level	Non-migrant, *n* = 36,702	Migrant, *n* = 6,605	*p*-Value
Sex	Male	15,891 (43.3%)	2,823 (42.7%)	0.40
	Female	20,811 (56.7%)	3,782 (57.3%)	

Socioeconomic status	Low	3,588 (9.8%)	1,177 (17.9%)	<0.001
	Middle	20,651 (56.4%)	3,601 (54.6%)	
	High	12,403 (33.8%)	1,815 (27.5%)	

Health insurance	Statutory	29,993 (81.7%)	5,867 (88.8%)	<0.001
	Private or others	6,709 (18.3%)	738 (11.2%)	

Living in a partnership	Yes	22,731 (62.2%)	3,955 (60.1%)	0.001
	No	13,799 (37.8%)	2,622 (39.9%)	

Place of residence	East	7,409 (20.2%)	717 (10.9%)	<0.001
	West	29,293 (79.8%)	5,888 (89.1%)	

Urbanity	Urban	25,014 (68.6%)	5,279 (81.0%)	<0.001
	Rural	11,423 (31.4%)	1,236 (19.0%)	

Social support	Weak	4,618 (13.0%)	1,152 (18.2%)	<0.001
	Moderate	17,718 (49.9%)	3,212 (50.8%)	
	Strong	13,173 (37.1%)	1,960 (31.0%)	

Age	18–24 years	3,669 (10.0%)	1,132 (17.1%)	<0.001
	25–29 years	2,143 (5.8%)	675 (10.2%)	
	30–34 years	2,363 (6.4%)	737 (11.2%)	
	35–39 years	3,121 (8.5%)	736 (11.1%)	
	40–44 years	4,410 (12.0%)	704 (10.7%)	
	45–49 years	4,206 (11.5%)	594 (9.0%)	
	50–54 years	3,482 (9.5%)	467 (7.1%)	
	55–59 years	3,267 (8.9%)	419 (6.3%)	
	60–64 years	2,634 (7.2%)	370 (5.6%)	
	65–69 years	2,916 (7.9%)	315 (4.8%)	
	70–74 years	2,301 (6.3%)	248 (3.8%)	
	75–79 years	1,118 (3.0%)	119 (1.8%)	
	80–84 years	726 (2.0%)	65 (1.0%)	
	85+ years	346 (0.9%)	24 (0.4%)	

Migrants were at an about 36% lower chance of utilizing regular dental checkups than non-migrants, corresponding to an 8% point lower likelihood of utilization (OR = 0.64; AME = −0.081). Differences are partly explained by the influence of predisposing and enabling factors (OR = 0.69; AME = −0.065). Younger age, being male, lower SES, a non-statutory health insurance, and poor social support were associated with poor utilization of regular dental checkups (Table 3). Also, individuals who did not live in a relationship, who resided in the Western part of Germany, and who lived in an urban setting were at a lower chance of utilizing regular dental checkups.

**Table 3:** Multivariable logistic regression model with utilization of dental checkups in the previous 12 months as the dependent variable.

Factor	OR	95% CI	AME	95% CI
Migrant (Ref: non-migrant)	0.69	0.64; 0.73	−0.064	−0.076; −0.053
Age	0.98	0.98; 0.99	−0.003	−0.004; −0.001
Female sex (Ref: male)	1.91	1.82; 2.00	0.106	0.098; 0.114
SES (Ref. low)				
Middle	1.78	1.65; 1.91	0.109	0.095; 0.124
High	2.61	2.40; 2.84	0.167	0.152; 0.183
Private health insurance (Ref: statutory)	0.75	0.70; 0.80	−0.048	−0.060; −0.036
Living in a partnership (Ref: not living in partnership)	1.63	1.55; 1.71	0.081	0.072; 0.089
Place of residence in Western Germany (Ref: Eastern Germany)	0.85	0.80; 0.91	−0.025	−0.035; −0.015
Living in an urban setting (Ref: Living in rural setting)	0.91	0.86; 0.96	−0.015	−0.023; −0.006
Social support (Ref: weak)				
Moderate	1.25	1.17; 1.34	0.038	0.026; 0.050
Strong	1.38	1.29; 1.49	0.054	0.041; 0.067

*ORs, odds ratios; AMEs, average marginal effects, including 95% confidence intervals (95% CI). No interaction effects included (German Health Update 2009/2010 survey, n = 41,220)*.

As an inspection of interaction effects based on marginal effects shows, respondents with a private/other health insurance were less likely to utilize this form of dental prevention among non-migrants, whereas no differences between both types of health insurance with respect to their relevance for the utilization of dental checkups could be observed for migrants. In the case of age, also the direction of the association differed. Although older individuals without migration background were less likely to utilize dental checkups than younger individuals, it were older individuals among migrants who were more likely to utilize this form of dental prevention than younger respondents (Table 4). No other interaction effects were significant and hence are not reported.

**Table 4:** Results of an interaction analysis regarding differing associations of predisposing/enabling factors and utilization of regular dental checkups between migrants and non-migrants.

	Non-migrants		Migrants	
	AME	95% CI	AME	95% CI
Age	−0.004	−0.005; −0.003	0.006	0.003; 0.01
Private health insurance (ref: statutory)	−0.05	−0.07; −0.04	−0.002	−0.04; 0.03

*AMEs, average marginal effects, including 95% confidence intervals (95% CI). Only significant interactions presented (German Health Update 2009/2010 survey, n = 41,220)*.

## Discussion

The aim of the present study was to examine enabling and predisposing factors for the utilization of preventive dental health care in migrants and non-migrants in Germany based on data from two national telephone surveys. The study identifies different enabling and predisposing factors that are relevant for the utilization of dental checkups among the population in Germany. The findings are in line with those of previous research which has been conducted on the utilization of dental services in other countries. For example, an investigation from Denmark has also found that females and individuals living in a partnership have a higher likelihood of utilizing dental prevention (12). Similarly, a higher SES has been found to increase the chance for preventive measures in general (35, 36) and for utilizing dental prevention in particular (16, 19, 37). In our study, individuals with a private instead of a statutory health insurance were at a lower chance of utilizing dental prevention. This may be related to differences in copayment and reimbursement agreements (38). Differences were also observed with respect to place of residence and type of residence area. Studies have shown that although there has been a convergence of the prevalence of oral health impairment and the utilization of dental health services between East and West Germany, regular dental visits are still more common among East German adults (39, 40). Similar findings have been documented for German children (41). A recent study found increasing geographical differences in the ratio of dental service demand and supply, with few clusters of overserviced units in or around urban areas compensating demand for larger numbers of underserviced areas (supporting central place theory) (42).

Our study also reveals that migrants utilize dental checkups less frequently than non-migrants. Differences are only partially explained by the different enabling and predisposing factors which we were able to take into account as a multivariable analysis adjusting for these factors shows. This corresponds to findings from other studies (9–12) and suggests that additional factors associated with migration status need to be considered when addressing differences in the utilization of dental prevention. Access to dental prevention may be, similar to access to prevention in general, limited by factors on the patient, provider, and system level. Factors on the patient level comprise, for example, disadvantageous perceptions and beliefs of health and illness and a low health literacy (28, 43, 44). These could be relevant need and predisposing factors as conceptualized by the Behavioral Model of Health Services Use (17, 18). Factors on the provider and system level include among others a poor cultural and migrant sensitivity of services (3, 44, 45). Future studies need to explore which patient-, provider-, and system-level actors are most relevant for the low utilization of dental prevention in migrants.

Although most predisposing and enabling factors did not significantly differ between migrants and non-migrants, the study showed that considerable age-related differences existed. While higher age was associated with a decreasing likelihood for the utilization of dental prevention in non-migrants, the association was reversed in migrants. Since there was no information on the time of immigration available in the data set and no differentiation between individuals who migrated themselves and those with immigrant parents was made, this effect at least in part could be related to acculturation processes and increased familiarity with the German health system (46).

The negative effect of a private insurance on the utilization of dental prevention could only be observed for non-migrants. A negative association between having a private insurance and using dental checkups has been found among non-migrant children and may be due to differences in cost, since dental prevention is covered by the statutory health insurance, but not necessarily by private health insurance contracts (47). The fact that a lower chance could only be observed for migrants could be related to differences between migrants and non-migrants in willingness to pay for dental checkups. This assumption, however, has to be verified by further studies.

Strengths of the analysis are the large size of the sample and the high quality of the gathered information. There are also some limitations in our study which need to be considered. The study was designed to reach adults with a landline phone, excluding individuals with only mobile or no phone at all. Given that in 2011 approximately 92.7% of German households had a landline connection, this could potentially have resulted in a bias toward individuals with a higher age and lower SES, among whom landline coverage was higher (48, 49). However, as the proportion of individuals who do not have a landline phone is rather small, we consider this bias to be of minor influence. This assumption is also supported by official statistics that show that in terms of the distribution of demographic and socioeconomic factors, the sample is similar to that of the total population in Germany (50). The inclusion criterion of high proficiency of the German language may have led to migrants being underrepresented in the study and to underestimating the differences between both population groups. The data we use were collected in 2009/2010 and are therefore slightly dated. Although studies on disparities between migrants and non-migrants with respect to other preventive services did not identify significant variation over time (51), investigations based on more recent data need to examine whether this is also true for dental prevention. Our data are based on self-report. Given that the time frame the question on the utilization of dental checkups referred to is rather small (12 months), we do not consider a recall bias to have distorted our findings. We also consider self-reported information on demographic and socioeconomic factors to be valid given that their distribution in our sample is similar to that of the total population in Germany (50). While we were able to take into account some enabling and predisposing factors, need factors such as the perceptions of health and illness and the respondent’s appraisal of the necessity to use dental health care could not be considered as these information were not available in the secondary data set we used. The Andersen model had been shown to provide a valuable framework for the study of the utilization of health care in different settings and among different population groups. Using this framework (13), future studies should also examine personal health practices as well as barriers migrants experience in the dental health care system and that need to be considered in order to implement patient-oriented services and to reduce disparities in access to dental care.

## Ethics Statement

The survey data collected by the Robert Koch Institute fulfill all necessary requirements and guidelines of the Federal data protection act. The survey was voluntary, anonymous and did not include experiments. Therefore, no further ethical approval was necessary.

## Author Contributions

PB, FE, and DW developed the concept and design of the study. PB performed the statistical analysis, interpreted the findings, and drafted the manuscript. FE and DW helped with the statistical analysis and data interpretation. All the authors read and approved the final manuscript.

## Conflict of Interest Statement

The authors declare that the research was conducted in the absence of any commercial or financial relationships that could be construed as a potential conflict of interest.
